# Anti-Jamming Resource-Allocation Method in the EH-CIoT Network through LWDDPG Algorithm

**DOI:** 10.3390/s24165273

**Published:** 2024-08-14

**Authors:** Fushuai Li, Jiawang Bao, Jun Wang, Da Liu, Wencheng Chen, Ruiquan Lin

**Affiliations:** College of Electrical Engineering and Automation, Fuzhou University, Fuzhou 350108, China; fsli_16@163.com (F.L.); 17305911197@163.com (J.B.); wangjunfzu@fzu.edu.cn (J.W.); liuda_0625@163.com (D.L.); qwer159606wc@163.com (W.C.)

**Keywords:** EH-CIoT network, resource allocation, anti-jamming method, linearly weighted deep deterministic policy gradient

## Abstract

In the Energy-Harvesting (EH) Cognitive Internet of Things (EH-CIoT) network, due to the broadcast nature of wireless communication, the EH-CIoT network is susceptible to jamming attacks, which leads to a serious decrease in throughput. Therefore, this paper investigates an anti-jamming resource-allocation method, aiming to maximize the Long-Term Throughput (LTT) of the EH-CIoT network. Specifically, the resource-allocation problem is modeled as a Markov Decision Process (MDP) without prior knowledge. On this basis, this paper carefully designs a two-dimensional reward function that includes throughput and energy rewards. On the one hand, the Agent Base Station (ABS) intuitively evaluates the effectiveness of its actions through throughput rewards to maximize the LTT. On the other hand, considering the EH characteristics and battery capacity limitations, this paper proposes energy rewards to guide the ABS to reasonably allocate channels for Secondary Users (SUs) with insufficient power to harvest more energy for transmission, which can indirectly improve the LTT. In the case where the activity states of Primary Users (PUs), channel information and the jamming strategies of the jammer are not available in advance, this paper proposes a Linearly Weighted Deep Deterministic Policy Gradient (LWDDPG) algorithm to maximize the LTT. The LWDDPG is extended from DDPG to adapt to the design of the two-dimensional reward function, which enables the ABS to reasonably allocate transmission channels, continuous power and work modes to the SUs, and to let the SUs not only transmit on unjammed channels, but also harvest more RF energy to supplement the battery power. Finally, the simulation results demonstrate the validity and superiority of the proposed method compared with traditional methods under multiple jamming attacks.

## 1. Introduction

With the development of information technology and machine-to-machine communication, more and more physical devices are connecting to the Internet. Thus, the Internet of Things (IoT) came into being. The IoT plays an important role in fields such as transportation, healthcare, industrial automation and disaster response [1]. However, the huge number of the IoT devices generate large amounts of exchanging data, which increases the demand for spectrum resources.

Due to the scarcity of spectrum resources, improving spectrum utilization is an important issue [2]. Cognitive Radio (CR) is an effective tool to alleviate this problem. CR allows the Secondary Users (SUs) to opportunistically access the spectrum band for data transmission by sensing the activities of the Primary Users (PUs) [3]. In the CR system, the SUs have two access modes: interweave and underlay. In interweave mode, the SUs can only access the spectrum bands that are not occupied by the PUs. In underlay mode, the SUs can access the spectrum bands occupied by the PUs, but they must limit the transmission power to ensure that the interference to the PUs is less than the threshold. The Cognitive Internet of Things (CIoT) is the combination of CR and IoT. It gives cognitive abilities to the IoT devices. And CIoT is receiving increasing attention [4].

Most IoT devices are powered by batteries. The limited battery capacity is a major problem restricting the development of IoT. Energy-Harvesting (EH) can alleviate the problem of energy constraint [5]; it can harvest energy from wind, heat, light, and RF signals. Therefore, there has been a lot of interest in harvesting RF energy from the environment [6], and the impacts of sensing energy and data availability on the secondary throughput of energy harvesting cognitive radio networks have received attention from researchers [7]. Combining CR technology with RF energy harvesting and IoT called Energy-Harvesting Cognitive Internet of Things (EH-CIoT); it can alleviate the spectrum scarcity caused by the rapid growth of IoT devices and energy scarcity caused by battery capacity restrictions. This paper considers the uplink EH-CIoT network model.

Protecting EH-CIoT networks from malicious attacks is a prominent research area. Due to the broadcast nature, the EH-CIoT network is susceptible to security risks. Attacks in the EH-CIoT network include spectrum-sensing data falsification, eavesdropping, primary user emulation, and jamming attacks [8,9]. Among these, jamming attacks are regarded as the most frequent and threatening ones since they can reduce the throughput of transmission, paralyze a network, or interrupt the communication service [10,11].

## 2. Related Works

In recent years, many studies have considered the EH-CIoT network, and have proposed different transmission algorithms. In [12], the authors proposed a mixed integer linear programming method to increase the EH-CIoT network throughput while ensuring that each node met the minimal energy demand and the quality of service. In [13], the authors considered a spectrum-sharing scheme for simultaneous information decoding under the EH-CIoT network overlay mode. They proposed a method to improve the total throughput and Energy Efficiency (EE) by measuring the typical peer-to-peer transmission time. In [14], the authors proposed an EH-CIoT optimization method to find an equilibrium between Spectral Efficiency (SE) and EE. The throughput performance of the EH-CIoT network communication devices was studied in [15]. The authors calculated throughput by computing several network metrics linked to the EH-CIoT network.

Due to the broadcast nature of the wireless communication, the EH-CIoT network is susceptible to jamming attacks. Therefore, many studies are dedicated to anti-jamming. In [16], the authors studied the channel-allocation problem under active and passive jamming attacks, and they proposed a probability-based channel-management mechanism to defend against jamming attacks. In [17], the authors proposed a multi-armed bandit strategy and an anti-jamming game to enhance the wireless communication service of the SUs. However, it was short-sighted and only focused on the optimal throughput performance of the current timeslot, but ignored LTT utility. In [18], the authors applied the future IoT laboratory testbed to capture the realistic radio propagation environment more accurately. They evaluated the effectiveness of the system’s spectrum-management strategy under jamming attacks. Because of latency sensitivity, in [19], the authors proposed a batch-based, security-aware MAC protocol. This protocol enabled the SUs to have jamming-sensing capability while the transmission was in parallel, aiming to improve network service. Although the above studies achieved success in anti-jamming, the proposed methods all require prior knowledge such as statistics information of PUs or jammers. But in a highly dynamic environment of the EH-CIoT network, it is unrealistic to acquire complete prior knowledge, such as accurate channel state information and activity state information of the PUs, etc. In addition, the offline models typically require accurate datasets for training and need to be retrained when new data are added. Therefore, its ability to cope with the dynamic EH-CIoT network is weak.

Reinforcement Learning (RL) is believed to help achieve optimal communication service strategies for the EH-CIoT network, which uses agents to continuously interact with the environment without prior knowledge to learn strategies. In [20], the authors proposed a novel channel-management method based on Q-learning to defend against jamming attacks. In [21], the authors proposed a joint power and channel-management method based on event-driven Q-learning to adaptively reduce jamming and increase throughput. In wireless communication networks, the problems of resource allocation and jamming mitigation will produce a large state and action space, and the traditional Q-learning algorithms are ineffective and hard to converge. In [22], the authors proposed a fast Deep Q-Learning (DQN)-based anti-jamming method, which effectively improved the transmission signal-to-interference-plus-noise ratio. In [23], the authors applied the double DQN algorithm to the multi-user model, and they proposed a frequency hopping method to defend against jamming attacks. In [24], to solve the problem of continuous power control in continuous state space and action space, the authors proposed an Actor-Critic DQN method to allocate transmission power more accurately. In [25], the authors proposed a multi-agent DRL cooperative resource-allocation method to manage the member grouping and power for the EH-CIoT network. In [26], the authors proposed a Deep Deterministic Policy Gradient (DDPG) method to improve the Long-Term Throughput (LTT) of the energy-constrained CR-NOMA network by optimizing the time-sharing coefficient and the SUs’ transmission power. In [27], the authors studied an optimal transmission algorithm in the EH-CIoT network; they used a DDPG algorithm to handle dynamic uplink access and continuous power control, which could effectively improve the LTT of the EH-CIoT network. In [28], the authors tackled the tradeoff between the active probability and spectrum access opportunity; they derived the optimal final decision threshold that maximizes the expected achievable throughput of the EH cognitive radio network. In [29], the authors exploited the optimal time allocation between PUs and SUs, and balanced the tradeoff between energy harvesting and packet transmission to obtain the maximum total achievable throughput. In [30], the authors proposed the Adjusted-Deep Deterministic Policy Gradient (A-DDPG) and combination of A-DDPG and convex optimization method to effectively improve the long-term throughput of the ambient backscatter communications and radio frequency-powered cognitive radio network by jointly controlling the time scheduling and energy management of SUs.

With the upgrading of jammers, wireless communication devices are prone to several types of intelligent jamming attacks. Therefore, some studies focus on defending against intelligent jamming attacks. In [31], the authors proposed a hierarchical reinforcement learning-based hybrid hidden strategy to defend against intelligent reactive jamming attacks. In [32], the authors considered the intelligence full-duplex jammers, which could maximize the utility of eavesdropping and jamming by optimizing the jamming power. The authors proposed a Bayesian–Stackelberg Game method to defend the intelligence full-duplex jammers and effectively improve the utility of legitimate communication devices. In [33], the author proposed a two-layer reception strategy to defend the jamming attacks caused by attackers using reconfigurable intelligent surface on multiple users.

## 3. Contribution and Organization

The EH-CIoT network is widely studied because it can effectively alleviate the problems of spectrum scarcity and energy constraint. In addition, the broadcast nature of the EH-CIoT network makes it vulnerable to jamming attacks. Therefore, studying the anti-jamming method of the EH-CIoT network is an important and practical problem. The main works of this paper are summarized in the following:

- This paper considers the EH-CIoT multi-user transmission network under jamming attacks, and deploys Agent Base Station (ABS) to reasonably allocate resources for multiple SUs to maximize the LTT of the network.
- This paper models the proposed problem as a Markov Decision Process (MDP) without prior knowledge, and proposes a Linearly Weighted Deep Deterministic Policy Gradient (LWDDPG) algorithm that enables the ABS to effectively learn resource-allocation strategies in the process of interacting with the dynamic environment. The proposed method enables the ABS to reasonably allocate transmission channels, continuous power, and work modes to the SUs.
- This paper carefully designs the reward function. Specifically, this paper proposes to take the throughput and RF energy harvested by the SUs as the two-dimensional rewards, which can enable the ABS to evaluate its actions more effectively and guide the ABS to make strategies that are beneficial for maximizing the LTT of the EH-CIoT network.

The remainder of this paper is organized as follows. First, Section 4 and Section 5 propose the EH-CIoT model and the jamming models, respectively. Second, an MDP-based optimization problem is formulated in Section 6. Third, Section 7 proposes the LWDDPG RL-based algorithm for an interweave EH-CIoT under jamming attacks and gives the detailed steps of the algorithm. Finally, Section 8 analyzes the simulation results and Section 9 presents the conclusion.

## 4. System Model

**Figure 1:**
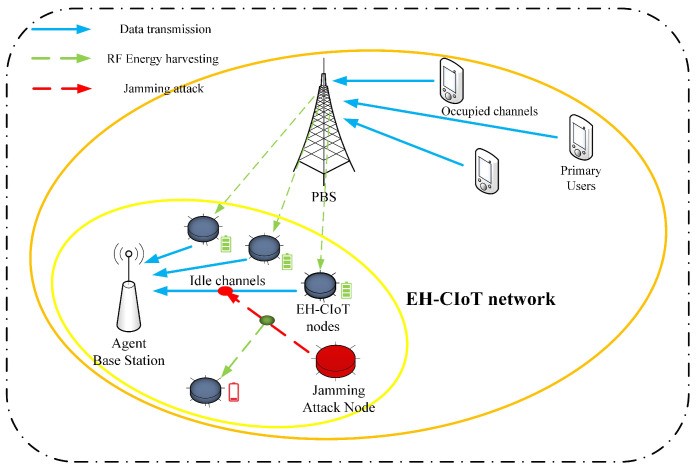
System model.

In specific application scenarios, the system model shown in Figure 1 can be a wireless sensor network used for environmental monitoring. The nodes with energy-harvesting capability monitor real-time environmental data and transmit them to base stations.

This paper considers the interweave mode of the CR [27]. Figure 1 shows the multi-user EH-CIoT network under jamming attacks. The EH-CIoT network is composed of a Primary Users Network (PUN) which has *M* PUs and a Primary Base Station (PBS), one ABS and *N* SUs, and a Jamming Attack Node (JAN). Due to the fact that the SUs in the EH-CIoT network are IoT devices and have EH function, this paper refers to the SUs as the EH-CIoT nodes. In this EH-CIoT network, the PUN has *K* licensed channels. The PUs use licensed channels to communicate with the PBS. At the beginning of each timeslot, the ABS performs spectrum sensing to identify the idle licensed channels. This paper assumes the ABS can always obtain perfect spectrum-sensing results [34]. Due to the uncertainty of the PU activities, the number of the idle licensed channels IK(t) may vary in each timeslot *t*. $I_{k}\left(t\right)=\left\{1\left(\mathrm{idle}\right),0\left(\mathrm{busy}\right)\right\}$ represents the state of the *k*-th licensed channel sensed by the ABS at the timeslot *t*, and $IK\left(t\right)=\sum_{k=1}^{K}I_{k}\left(t\right)$.

### 4.1. Channel Gain

In the EH-CIoT network, both small-block Rayleigh fading and large-scale path loss fading are considered [35]. And this paper considers the channel gain to be changing between varies timeslots. Therefore, the channel gain $g_{xy}\left(t\right)$ at the *t*-th timeslot is described as:

(1) $$g_{xy}\left(t\right)={\left(\frac{d_{xy}}{d_{0}}\right)}^{-\alpha }\cdot {\left|h_{xy}\left(t\right)\right|}^{2}$$

where $h_{xy}\left(t\right)$ denotes the small-block Rayleigh fading, ${\left(\frac{d_{xy}}{d_{0}}\right)}^{-\alpha }$ represents the large-scale pathloss fading, $d_{xy}$ represents the distance between *x* and *y*, x∈{[1,2,…,N],P,J} denotes the *x*-th transmitter (with the number of EH-CIoT nodes *N*, PBS and JAN), y∈{[1,2,…,N],b} denotes the *y*-th receiver (with the number of EH-CIoT nodes *N* and ABS), α represents the path loss index and $d_{0}$ represents the reference distance $\left(d_{xy}>d_{0}\right)$ [36]. Therefore, $g_{ib}\left(i=1,2,\ldots ,N\right)$ represents the channel gain from the *i*-th EH-CIoT node to the ABS; $g_{Pi}$ represents the channel gain from the PBS to the *i*-th EH-CIoT node; $g_{si}\left(s=1,2,\ldots ,N\right)$ represents the channel gain between *s*-th and *i*-th EH-CIoT nodes, and s≠i; $g_{Ji}$ represents the channel gain from JAN to the *i*-th EH-CIoT node and $G\left(t\right)=\left\{g_{ib}\left(t\right),g_{Pi}\left(t\right),g_{si}\left(t\right),g_{Ji}\left(t\right)\right\}$ is the set of the channel gains.

### 4.2. Two Mode Selections for EH-CIoT Nodes

In the EH-CIoT network, each EH-CIoT node has the same configuration: a single antenna, a rechargeable battery, a transmitter, a receiver, and an energy-acquisition device. The EH-CIoT nodes can only perform RF energy harvesting or transmission in each timeslot. The energy harvested in the current timeslot will not be used immediately but stored in the rechargeable battery. Due to the limitation of spectrum resources, one EH-CIoT node can only access one idle licensed channel. Due to the scarcity of spectrum resources and the fact that massive IoT devices access wireless networks [29], like [27], this paper considers that all the EH-CIoT nodes always have the data that need to be transmitted.

Assume that all the EH-CIoT nodes are managed by the ABS. The EH-CIoT node sends its battery level state set B(t) (see Section 4.3 for details) to the ABS through a dedicated control channel at the beginning of each timeslot *t*. As the core of the EH-CIoT network, the ABS determines the work mode (harvesting mode or transmission mode), channel access and transmission power of all EH-CIoT nodes in the current timeslot *t* according to IK(t), the channel gain information set G(t), the received battery level state set B(t) and the previous slot received result set Y(t−1) (see Section 6 for details). Then, the resource-allocation decisions are broadcast to all EH-CIoT nodes. Let $P_{i}^{C}\left(t\right)$ represent the transmission power of the *i*-th EH-CIoT node (i=1,2,…,N) in *t*-th timeslot, and $P_{max}^{C}$ denote the maximum transmission power of the EH-CIoT nodes. Therefore, in the *t*-th timeslot, the continuous power-allocation set of all EH-CIoT nodes is expressed as $P\left(t\right)=\left\{P_{1}^{C}\left(t\right),P_{2}^{C}\left(t\right),\ldots ,P_{N}^{C}\left(t\right)\right\}$. If $P_{i}^{C}\left(t\right)>0$, the *i*-th EH-CIoT node works in transmission mode with power $P_{i}^{C}\left(t\right)$. If $P_{i}^{C}\left(t\right)=0$, the *i*-th EH-CIoT node works in harvesting mode. The work mode $M_{i}^{C}\left(t\right)$ of the *i*-th EH-CIoT node in the *t*-th timeslot can be described as:

(2) $$M_{i}^{C}\left(t\right)=\left\{\begin{array}{cc} 0\;(\mathrm{harvesting}\;\mathrm{mode}), & \;\mathrm{if}\;P_{i}^{C}\left(t\right)=0\\ 1\;(\mathrm{transmission}\;\mathrm{mode}), & \;\mathrm{if}\;P_{i}^{C}\left(t\right)>0 \end{array}\right.$$

**Figure 2:**
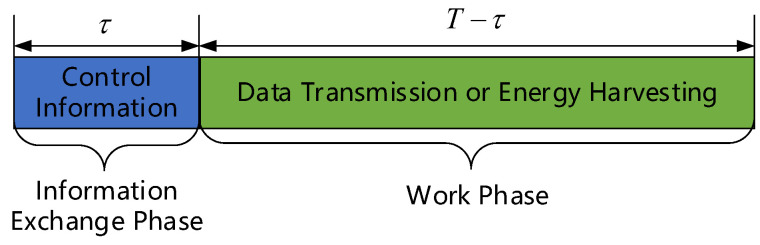
Timeslot structure.

This paper lets $M\left(t\right)=\left\{M_{1}^{C}\left(t\right),M_{2}^{C}\left(t\right),\ldots ,M_{N}^{C}\left(t\right)\right\}$ as the work mode set of all EH-CIoT nodes in the *t*-th timeslot. The number of EH-CIoT nodes that select the transmission mode is IC(t), and IC(t)≤IK(t). The timeslot structure is shown in Figure 2, where *T* is the length of a timeslot, and τ is the length of the information-exchange phase in a timeslot.

In the EH-CIoT network, it is assumed that the energy consumed in the information-exchange phase is fixed and represented by $e_{f}$. If the remaining energy of the EH-CIoT node is insufficient to complete the information exchange, it will not be able to send its battery state. When the ABS does not receive the battery state information of the EH-CIoT node in the information-exchange phase, it will consider the EH-CIoT node in a low-power state and control it to enter harvesting mode.

### 4.3. Energy Harvesting and Renewal

In the EH-CIoT network, the PBS, the JAN, and the ABS are powered by the grid. The EH-CIoT nodes are powered by rechargeable batteries, and they use EH technology to charge the batteries.

(1) **Energy Harvesting**. This paper considers that the EH-CIoT nodes can harvest energy from three kinds of transmission signals: the PUs, the JAN, and other EH-CIoT nodes. Since the RF energy harvester typically operates over a range of frequencies [37], this paper considers that the EH-CIoT nodes can only harvest energy from one channel. The transmission power of the PU in the *k*-th licensed channel is $P_{k}^{p}\left(k=1,2,\ldots ,K\right)$. The jamming power of the JAN in the *k*-th licensed channel is $P_{k}^{J}\left(t\right)$.

This paper uses the linear energy-harvesting model. Therefore, the RF energy harvested by *i*-th EH-CIoT nodes in the *t*-th timeslot can be expressed as:

(3) $$\begin{array}{c} E_{i}^{C}\left(t\right)=\eta \left(T-\tau \right)\left(1-M_{i}^{C}\left(t\right)\right)\cdot P\left(t\right)g\left(t\right) \end{array}$$

where η is the energy-conversion rate. P(t) represents the transmission power, and $P\left(t\right)\in \left\{P_{k}^{p}\left(t\right),P_{k}^{J}\left(t\right),P_{s}^{C}\left(t\right)\right\}$. Correspondingly, g(t) represents channel gain, and $g\left(t\right)\in \left\{g_{Pi}\left(t\right),g_{Ji}\left(t\right),g_{si}\left(t\right)\right\}$. Due to the different transmission power and channel gain in different channels, the EH-CIoT nodes obtain different energy through EH on different channels. The amount of energy harvested by the EH-CIoT nodes depends on the channels and work modes allocated to them by the ABS. The harvested energy set of all EH-CIoT nodes in the *t*-th timeslot is expressed as $E\left(t\right)=\left\{E_{1}^{C}\left(t\right),E_{2}^{C}\left(t\right),\ldots ,E_{N}^{C}\left(t\right)\right\}$. It is worth noting that even if multiple EH-CIoT nodes harvest energy on the same channel, the energy harvested by the EH-CIoT nodes will not be discounted.

(2) **Battery Update**. In the EH-CIoT network, the maximum battery capacity of the EH-CIoT nodes is $B_{\max}$. The rechargeable batteries are assumed to be ideal so that there is no energy loss during energy storage or recovery. The battery power cannot exceed the maximum capacity. The battery state set of all EH-CIoT nodes in the *t*-th timeslot is expressed as $B\left(t\right)=\left\{B_{1}^{C}\left(t\right),B_{2}^{C}\left(t\right),\ldots ,B_{N}^{C}\left(t\right)\right\}$. The evolution of the battery state of the *i*-th EH-CIoT node from the timeslot *t* to the timeslot t+1 can be expressed as:

(4) $$\begin{array}{c} B_{i}^{C}\left(t+1\right)=\min\left\{B_{\max},B_{i}^{C}\left(t\right)+E_{i}^{C}\left(t\right)-\left(T-\tau \right)M_{i}^{C}\left(t\right)P_{i}^{C}\left(t\right)-F_{i}\left(t\right)e_{f}\right\} \end{array}$$

where

(5) $$F_{i}\left(t\right)=\left\{\begin{array}{c} 0\;,\;\mathrm{if}\;B_{i}^{C}\left(t\right)\leq e_{f}\\ 1\;,\;\mathrm{if}\;B_{i}^{C}\left(t\right)>e_{f} \end{array}\right.$$

represents whether the *i*-th EH-CIoT node has enough energy to report the $B_{i}^{C}\left(t\right)$ to the ABS in the information-exchange phase.

## 5. Jamming Attack Models

In the EH-CIoT network, the JAN aims to reduce the throughput of the EH-CIoT nodes. This paper considers three types of jamming attacks, random jamming attacks, scanning jamming attacks, and intelligent reactive-scanning jamming attacks. In addition, this paper describes the strategies of the JAN as $G\left\{P^{J}\left(t\right),P^{J}\left(t\right)\right\}$, where the $P^{J}\left(t\right)$ is the jamming probability and $P^{J}\left(t\right)$ is the jamming power. Then, this paper describes $P^{J}\left(t\right)$ and $P^{J}\left(t\right)$ under different jamming attacks. This paper gives them appropriate subscripts under different jamming attacks.

**Figure 3:**
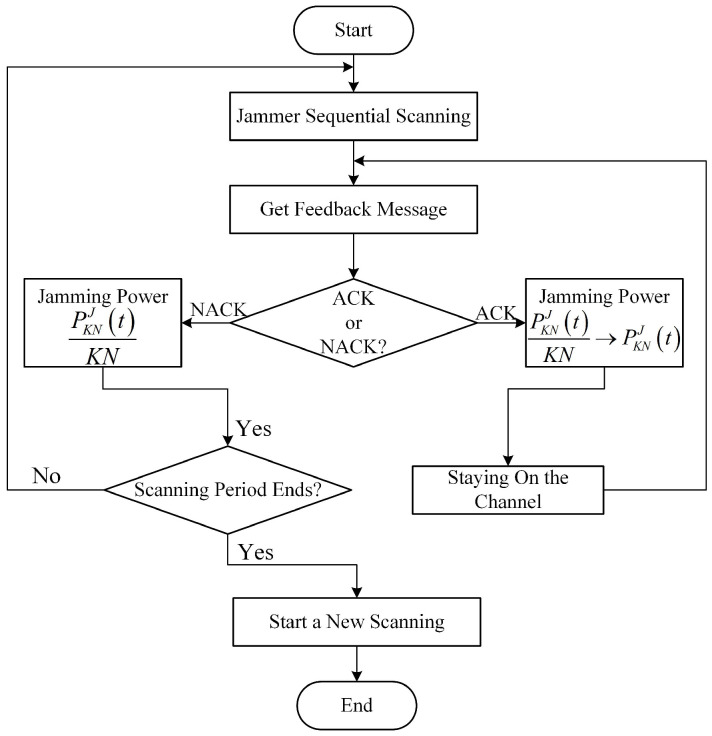
The flowchart of reactive-scanning jamming attack.

(1) **Random Jamming Attack**. The JAN jams the *k*-th channel with probability $P_{k}^{J}\left(t\right)$ in the *t*-th timeslot, and the jamming power is $P_{k}^{J}\left(t\right)$.

(2) **Scanning Jamming Attack**. The JAN jams $K_{N}$ channels with probability $P_{K_{N}}^{J}\left(t\right)$ in the *t*-th timeslot $\left(K_{N}\leq K\right)$, and the jamming power is $P_{K_{N}}^{J}\left(t\right)/K_{N}$, where $P_{K_{N}}^{J}\left(t\right)$ is the jamming power when the JAN jams one channel. In the next timeslot, the JAN chooses another $K_{N}$ channels to jam without repetition, i.e., the JAN needs $K/K_{N}$ timeslots to traverse *K* channels. This paper defines that the JAN finishes jamming with all *K* channels without repetition as a scanning period. Therefore, each scanning period contains $K/K_{N}$ timeslots.

(3) **Reactive-Scanning Jamming Attack**. Unlike scanning jamming attack, after the JAN allocates its jamming power $P_{K_{N}}^{J}\left(t\right)/K_{N}$ to $K_{N}$ channels, it senses the ACK/NACK message of the jammed channels (the ACK/NACK is a feedback message that the receiver acknowledges channel access results to the transmitter. When the receiver receives an ACK message, it represents successful transmission; otherwise, the transmission fails [38]). If the ACK is sensed, it means that although the JAN and the EH-CIoT node are accessed to the same channel, the jamming power is insufficient to prevent the EH-CIoT node’s data transmission. At this time, the JAN will change the jamming strategy from scanning attack to centralized attack, and transmit all the jamming power to the channel where ACK is located, i.e., $P_{K_{N}}^{J}\left(t\right)/K_{N}\to P_{K_{N}}^{J}\left(t\right)$. Until the NACK is sensed, it means that the JAN successfully prevents the EH-CIoT node’s data transmission. At this point, the JAN will leave the channel and begin the next timeslot jamming attack. If the current scanning period ends, the JAN will start a new scanning period. Figure 3 shows the flowchart of reactive-scanning jamming attack.

In each timeslot, the maximum jamming power of the JAN is $P_{\max}^{J}$. There is also a constraint on the time-averaged jamming power $P_{avg}^{J}=P_{K_{N}}^{J}\left(t\right)/K_{N}$, where $P_{avg}^{J}<P_{\max}^{J}$. The JAN can select the jamming power from a set of power levels $P_{J}\left(t\right)=\left\{P_{J0},P_{J1},\ldots ,P_{JM}\right\}$. Therefore, in the EH-CIoT network, the Signal to Interference plus Noise Ratio (SINR) of the *i*-th EH-CIoT node received by the ABS in a jamming attack environment can be expressed as:

(6) $${\mathrm{SINR}}_{i}\left(t\right)=\left\{\begin{array}{cc} \frac{P_{i}^{C}\left(t\right)g_{ib}\left(t\right)}{n}, & f_{i}^{k}\left(t\right)\neq f_{J}^{k}\left(t\right),M_{i}^{C}\left(t\right)=1\\ 0, & M_{i}^{C}\left(t\right)=0\\ \frac{P_{i}^{C}\left(t\right)g_{ib}\left(t\right)}{n+P^{J}\left(t\right)g_{Ji}\left(t\right)}, & f_{i}^{k}\left(t\right)=f_{J}^{k}\left(t\right),M_{i}^{C}\left(t\right)=1 \end{array}\right.$$

where $f_{i}^{k}\left(t\right)=f_{J}^{k}\left(t\right)$ represents the channel accessed by *i*-th EH-CIoT node which is the same as the channel attacked by the JAN, and $n∼N\left(0,\omega^{2}\right)$ represents the additive Gaussian white noise. In (6), when the work mode of the EH-CIoT node is in harvesting mode, no transmission power is generated; the SINR is 0. When the work mode of the EH-CIoT node is transmission mode and it accesses the same channel as the JAN, the transmission of the EH-CIoT node will be severely jammed or even interrupted. Therefore, the ABS needs to reasonably allocate transmission channels, continuous power and work modes to the EH-CIoT nodes.

## 6. Problem Formulation

In the EH-CIoT network under jamming attacks, the ABS learns the attack strategy of the JAN through the signal reception status of the previous timeslot, predicts the channel that will be attacked, and arranges the EH-CIoT nodes that have low battery power to harvest energy, other EH-CIoT nodes transmit on the unjammed channels. The signal reception result of the previous timeslot in the information-exchange phase can be expressed as:

(7) $$\begin{array}{c} Y\left(t-1\right)=\left\{y_{1}^{A}\left(t-1\right),y_{2}^{A}\left(t-1\right),\ldots ,y_{N}^{A}\left(t-1\right)\right\} \end{array}$$

and

(8) $$y_{i}^{A}\left(t-1\right)=\left\{\begin{array}{c} 0,NACK\\ 1,ACK \end{array}\right.$$

That is, ACK: the ABS successfully receives the EH-CIoT node transmission data; NACK: the ABS fails to receive the EH-CIoT node transmission data or the EH-CIoT node is performing energy harvesting.

Considering the long-term system performance of the EH-CIoT network, the goal of this paper is to maximize the LTT of the EH-CIoT network under jamming attacks. The instant throughput of the EH-CIoT network in a timeslot can be calculated by Shannon’s capacity formula [39,40]:

(9) $$r_{t}^{A}=W\sum_{i=1}^{N}M_{i}^{C}\left(t\right)\left(T-\tau \right)\log\left(1+{\mathrm{SINR}}_{i}\left(t\right)\right)$$

where $r_{t}^{A}$ represents the instant throughput of the EH-CIoT network at the *t*-th timeslot and *W* represents the spectrum bandwidth.

Since the EH-CIoT network is energy constrained, it is not appropriate to simply maximize the instant throughput of the current timeslot, and future timeslots should also be considered. Therefore, the LTT of the EH-CIoT network at the *t*-th timeslot is calculated by the following formula:

(10) $$\begin{array}{cc} R^{A}\left(t\right) & =\sum_{v=t}^{\infty }\gamma^{v-t}r_{v}^{A}\\ \; & =W\sum_{v=t}^{\infty }\gamma^{v-t}\sum_{i=1}^{N}M_{i}^{C}\left(v\right)\left(T-\tau \right)\log\left(1+{\mathrm{SINR}}_{i}\left(v\right)\right) \end{array}$$

where 0<γ<1 represents the future discount rate. The problem of maximizing the LTT of the EH-CIoT network can be formulated:

(11) $$\max_{M_{i}^{C}\left(t\right),P_{i}^{C}\left(t\right)}E\left[R^{A}\left(t\right)\right]$$

(12) $$s.t.\;f_{i}^{k}\left(t\right)=\left\{0,1\right\}$$

(13) $$\;\;\;\;\;\;f_{J}^{k}\left(t\right)=\left\{0,1\right\}$$

(14) $$\;\;\;\;\;\;B_{i}^{C}\left(t\right)-F_{i}\left(t\right)e_{f}\geq \left(T-\tau \right)P_{i}^{C}\left(t\right)$$

(15) $$\;\;\;\;\;\;{\mathrm{SINR}}_{i}\left(t\right)\geq {\mathrm{SINR}}_{\mathrm{threshold}\;}$$

(16) IC(t)≤IK(t)

where E[·] represents the expected value. The constraint (12) and (13) ensure that the state of the channel *k* accessed by the *i*-th EH-CIoT node and the JAN is a binary value. Constraint (14) ensures that the energy of the EH-CIoT node working in transmission mode does not exceed the available remaining energy. Constraint (15) ensures that the SINR is not less than the threshold. Constraint (16) ensures that the number of EH-CIoT nodes that select the transmission mode IC(t) does not exceed the number of idle channels.

In the EH-CIoT network, if the ABS can obtain the transition probability of the active state of the PUN, the attack probability, and the jamming power of the JAN in advance, Equation (11) can be solved using the offline method. However, it is unrealistic for the ABS to obtain such complete information. The Deep Reinforcement Learning (DRL) can solve the indeterminate polynomial resource allocation of the ABS [41].

## 7. DRL-Based Transmission-Optimization Algorithm

In this section, this paper analyzes the state parameters of the EH-CIoT network and builds the RL framework, and then briefly introduces the necessary principles of the RL. Finally, the LWDDPG resource-allocation algorithm for an interweave EH-CIoT under jamming attacks is proposed.

### 7.1. Framework of RL-Based EH-CIoT Network

The ABS is responsible for allocating transmission channels, continuous power, and work modes to the EH-CIoT nodes to maximize the LTT of the EH-CIoT network. The aim is to enable the ABS to effectively learn these strategies and make optimal decisions without prior knowledge. This paper constructs an environment model that maps the system model to the MDP’s interactive environment [42]. The MDP consists of a quintuple, namely $MDP=\left(S,A,P_{sa},R,\gamma \right)$, where S is the state space, A is the action space, $P_{sa}$ is the state transition probability. Considering the proposed EH-CIoT dynamic environment model without prior knowledge, the $P_{sa}$ is unknown. R is the reward function, and γ is a discount factor that exponentially discounts the value of future rewards in (10). The agents use a discount factor to adjust the value placed on future rewards. The setting of state space, action space, and reward in the model is explained as follows.

**Agent**: The ABS in the EH-CIoT network. It interacts with the EH-CIoT environment under jamming attacks without prior knowledge to discover which actions yield the greatest rewards in certain situations. Compared with self-management of EH-CIoT nodes, setting the ABS as the agent and managing all EH-CIoT nodes is conducive to making global optimal decisions.

**State space** S: The ABS needs to collect real-time information of all EH-CIoT nodes at the beginning of each timeslot, perform spectrum sensing, obtain channel gain, and count information reception. Then the state space at the *t*-th timeslot is defined as:

(17) $$S_{t}=\left\{Y\left(t-1\right),I\left(t\right),G\left(t\right),B\left(t\right)\right\}$$

where $I\left(t\right)=\left\{I_{1}\left(t\right),I_{2}\left(t\right),\ldots ,I_{K}\left(t\right)\right\}$ represents the channel state set of the PUN, G(t) represents the channel gain set, B(t) represents the battery level set of all EH-C nodes and Y(t−1) represents the information reception set of the previous timeslot.

**Action space** A: The action of the *t*-th timeslot is defined as $A_{t}=\left\{M\left(t\right),P\left(t\right),C\left(t\right)\right\}$. Where $C\left(t\right)=\left\{C_{1}\left(t\right),C_{2}\left(t\right),\ldots ,C_{N}\left(t\right)\right\}$ represent the transmission channel set and let $C_{i}\left(t\right)$ represent the channel that the ABS allocates to the *i*-th EH-CIoT node. Overall, the ABS allocates transmission channels, continuous power, and work modes to all EH-CIoT nodes.

**Reward** R: When the ABS interacts with the environment, it uses the reward to evaluate the effectiveness of its action, estimate the distribution of states, and comprehend the surroundings. Therefore, a well-thought-out reward system is essential for the ABS to learn more efficiently. After taking an action in a state, the ABS will receive a reward $R\left(S_{t},A_{t}\right)$. Then, the system will move to the next state $S_{t+1}$.

To better guide the ABS to rationally allocate resources to the EH-CIoT nodes to maximize the LTT of the EH-CIoT network and realize anti-jamming, this paper sets the reward R as a two-dimensional vector. The reward is designed as:

(18) $$R\left(S_{t},A_{t}\right)=\left[r_{t}^{A},r_{t}^{E}\right]$$

where $r_{t}^{A}$ denotes the instant throughput reward of the system at the *t*-th timeslot in (9). Whether the transmission channel is jammed, the channel gain situation, and the allocation of EH-CIoT nodes transmission power all affect the size of $r_{t}^{A}$. Therefore, the ABS can evaluate the effectiveness of its actions through $r_{t}^{A}$. In addition, the battery power of the EH-CIoT nodes is also closely related to LTT. The adequate battery power can increase the transmission throughput of the EH-CIoT nodes. As mentioned earlier, due to the differences in transmission power and channel gain on different channels, the energy harvested by the EH-CIoT nodes on different channels also varies. Thus, this paper sets $r_{t}^{E}$ as the energy reward, which represents the energy harvested by the EH-CIoT nodes. The expression of the certain reward in the *t*-th timeslot is:

(19) $$r_{t}^{E}=\sum_{i=1}^{N}E_{i}^{C}\left(t\right)$$

Compared to $r_{t}^{A}$, $r_{t}^{E}$ is small, which can effectively prevent the ABS from excessively attempting to obtain rewards through EH. Although it cannot be guaranteed that the battery power of the EH-CIoT nodes working in transmission mode is always full, the proposed method can enable the EH-CIoT nodes to harvest more energy for transmission than traditional methods during the EH phase through the design of energy reward. Through the two-dimensional reward function, the ABS can continuously interact with the environment to evaluate the effectiveness of its actions and find the optimal actions to maximize the LLT.

Unlike the scalar rewards of the traditional RL algorithm, this paper designs the rewards as a two-dimensional vector that includes both instant throughput rewards and energy rewards. To solve this two-dimensional vector, this paper uses the Linear Weighted (LW) method [43]. The method allocates appropriate weight coefficients to the elements in the vector according to their importance, and the sum of their products is used as a new objective function. Formally, the method aims to maximize the following function:

(20) $$h_{i}\left(x\right)=\max\sum_{i=1}^{m}w_{i}f_{i}\left(x\right)$$

where $w_{i}$ is a non-negative weight and $\sum_{i=1}^{m}w_{i}=1$. The weights $w_{i}$ are often taken as a constant. The proposed reward consists of instant throughput reward and energy reward; they have different weights, so the weighted sum of reward vector elements can be expressed as $r=R\omega^{T}=r_{t}^{A}\omega_{a}+r_{t}^{E}\omega_{e}$, where $\omega =\left[\omega_{a},\omega_{e}\right]$. In our setup, the weight parameter value is in the range [0,1]. This method converts the two-dimensional reward vector to a scalar to enable the ABS to intuitively evaluate the effectiveness of its actions.

In RL, after choosing action *a* according to the policy π, the expected return in state *s* is usually represented by state-action value or Q-value:

(21) $$Q^{\pi }\left(s,a\right)=E\left[R^{A}\left(t\right)|S_{t}=s,A_{t}=a\right]$$

where $R^{A}\left(t\right)$ is the future cumulative reward (10). Since the ABS obtains the action in a given state according to the policy π, the agent’s target is to constantly learn the optimal policy $\pi^{*}=\arg\max_{\pi }Q^{\pi }\left(s,a\right)$. To obtain the optimal policy $\pi^{*}$, the agent uses the Bellman function to recursively calculate the $r_{t}^{A}$. The Bellman function separates the Q-function into the discounted future reward and the reward [44], that is

(22) $$\begin{array}{cc} Q^{\pi }\left(s,a\right) & =E\left[R^{A}\left(t\right)|S_{t}=s,A_{t}=a\right]\\ \; & =E\left[r_{t}^{A}+\gamma Q^{\pi }\left(S_{t+1},A_{t+1}\right)|S_{t}=s,A_{t}=a\right] \end{array}$$

Traditional RL algorithms are not suitable for the huge state space and action space. For this reason, DRL introduces Deep Neural Networks (DNNs) for better nonlinear fitting of the value function. DQN uses the function Q(s,a∣θ) to represent the approximate computation of the value function, where θ is the parameter of the neural network. Although DQN can make good decisions for discrete and low-dimensional action space problems, it is not suitable for continuous action control problems. To solve this problem, the Deep Policy Gradient (DPG) algorithm is adopted and an Actor–Critic (AC) method is proposed. DQN and DPG algorithms are combined in a further proposed DDPG algorithm that is based on the AC framework. To handle the high-dimensional continuous optimization problem in the proposed system model, this paper provides a LWDDPG resource-allocation algorithm for an interweave EH-CIoT under jamming attacks.

### 7.2. Linearly Weighted Deep Deterministic Policy Gradient-Based Power-Allocation Algorithm

**Figure 4:**
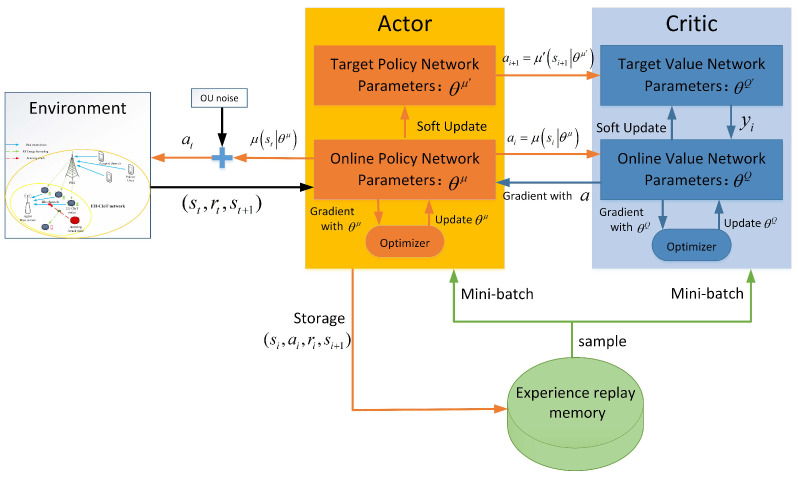
Framework of LWDDPG algorithm.

The framework of the LWDDPG algorithm is shown in Figure 4. It consists of three parts: actor policy network, critic value network, and experience buffer. The AC network includes four DNNs, that is, an Online Critic Network (OCN) with parameter $\theta^{Q}$, an Online Actor Network (OAN) with parameter $\theta^{\mu }$, a Target Critic Network (TCN) with parameter $\theta^{Q^{\prime }}$, and a Target Policy Network (TPN) with parameter $\theta^{\mu^{\prime }}$. The OAN $\mu \left(s|\theta^{\mu }\right)$ is used to build the mapping from states to actions, and the OCN $Q\left(s,a|\theta^{Q}\right)$ is used to estimate the value of actions. In the initialization phase, the TAN $\mu^{\prime }\left(s,a|\theta^{\mu^{\prime }}\right)$ and the TCN $Q^{\prime }\left(s,a|\theta^{Q^{\prime }}\right)$ are created by copying the parameters of the online network.

When updating network parameters, sample mini-batches from experience buffer *D* with capacity *C*. These mini-batches train the parameters through the gradient-descent method, then update the actor network, the critic network, and their corresponding target networks in turn. Any set of tuples sampled can be denoted as $\left(s_{x},a_{x},r_{x},s_{x+1}\right)$.

OCN is optimized by minimizing the loss between the target value and the Q function. The loss function of OCN can be formulated as the Mean Squared Error (MSE) of the difference as follows:

(23) $$L\left(\theta^{Q}\right)=E\left[{\left(y_{x}-Q\left(s_{x},a_{x}|\theta^{Q}\right)\right)}^{2}\right]$$

The target value $y_{x}$ is calculated as follows:

(24) $$y_{x}=r_{x}+\gamma Q^{\prime }\left(s_{x+1},\mu^{\prime }\left(s_{x+1}|\theta^{\mu^{\prime }}\right)|\theta^{Q^{\prime }}\right)$$

then use the gradient descent method to minimize $L\left(\theta^{Q}\right)$ to update the parameters in OCN.

For OAN optimization, its loss function can be obtained by summing the Q-functions of the states. OCN is used to calculate the evaluation value of the state action pair of OAN (the cumulative expected return), that is

(25) $$L\left(\theta^{\mu }\right)=E\left[Q\left(s_{x},\mu \left(s_{x}|\theta^{\mu }\right)|\theta^{Q}\right)\right]$$

then use the gradient ascent method to maximize $L\left(\theta^{\mu }\right)$ and update the parameters in OAN.

For the update of the two target networks, the DDPG algorithm adopts the soft update method, which can also be called exponential moving average, that is

(26) $$\;\mathrm{soft}\;\mathrm{update}:\;\left\{\begin{array}{c} \theta^{Q^{\prime }}\leftarrow \xi \theta^{Q}+\left(1-\xi \right)\theta^{Q^{\prime }}\\ \theta^{\mu^{\prime }}\leftarrow \xi \theta^{\mu }+\left(1-\xi \right)\theta^{\mu^{\prime }} \end{array}\right.$$

where ξ∈(0,1] represents the update rate of the target network.

By including noise in the actor policy, the exploration problem of ABS learning in continuous action spaces can be solved. Specifically, at each decision step, actions are chosen from a random process with expectation $\mu \left(s_{t}|\theta^{\mu }\right)$ and variance $\varepsilon \sigma^{2}$, namely $A_{t}∼N\left(\mu \left(S_{t}|\theta^{\mu }\right),\varepsilon \sigma^{2}\right)$, where ε is a parameter to attenuate the randomness of actions in the training process. The randomness of actions may lead to IC(t)>IK(t), which does not satisfy constraint (16). For this instability factor, the ABS will sort the SINR of the IC(t) nodes, select the IC(t) nodes with the largest SINR to allocate transmission mode, and allocate the harvesting mode for the others. The proposed LWDDPG resource-allocation algorithm for an interweave EH-CIoT under jamming attacks is given in Algorithm 1. This paper introduces energy rewards to enable the EH-CIoT nodes to harvest more energy to increase throughput, and uses the learning ability of the LWDDPG algorithm to enable the ABS to allocate transmission channels, continuous power, and work modes more reasonably for the EH-CIoT nodes; let the EH-CIoT nodes avoid transmitting on jammed channels and achieving anti-jamming. In the resource-allocation process of Algorithm 1, when all EH-CIoT nodes in the local area have insufficient energy and cannot harvest energy from the RF signals of other EH-CIoT nodes, they can still harvest the RF energy of the PUs and the JAN. And the proposed method can avoid this extreme situation through energy rewards and reasonable allocation of transmission power.
**Algorithm 1** LWDDPG Resource-Allocation Algorithm for the Interweave EH-CIoT Network Under Jamming Attacks.1:**Initialization:** Initialize online network parameters $\theta^{Q}$ and $\theta^{\mu }$ with random weights; Initialize the target network weights with $\theta^{Q}\to \theta^{Q^{\prime }}$ and $\theta^{\mu }\to \theta^{\mu^{\prime }}$; Empty experience buffer *D*; Initialize action random parameters ε; Initialize the battery level of the EH-CIoT nodes.2:**Iutput:** EH-CIoT network environment simulation parameters, JAN parameters and a weight vector $\omega =\left[\omega_{a},\omega_{e}\right]$.3:**for** episode = 1,2,…,F **do**4:  Initialize the environment.5:  Get the initial state $S_{0}$.6:  **for** t=1,2,…,G **do**7:   choose Action $A_{t}∼N\left(\mu \left(S_{t}|\theta^{\mu }\right),\varepsilon \sigma^{2}\right)$.8:   get scalar reward through $r=r_{t}^{A}\omega_{a}+r_{t}^{E}\omega_{e}$ and next state $S_{t+1}$.9:   Save data $\left(S_{t},A_{t},R_{t},S_{t+1}\right)$ to experience buffer *D*.10:  **if** *D* is full, **do**11:   Randomly sample transition data $\left(s_{x},a_{x},r_{x},s_{x+1}\right)$ of size $N_{B}$ from *D*.12:   Update OCN by minimizing $L\left(\theta^{Q}\right)$ in Equation (23)13:   Update OAN by maximizing $L\left(\theta^{\mu }\right)$ in Equation (25)14:   Soft update TCN and TAN by Equation (26).15:   Decay the action randomness: $\sigma^{2}\leftarrow \epsilon \sigma^{2}$16:  **end for**17:**end for** 18:**Output:** Optimal action $A_{t}$ of each timeslot.

## 8. Simulation Results

### 8.1. Simulation Settings

Through computer simulations, this paper evaluates the performance of the proposed LWDDPG resource-allocation algorithm. This paper simulates a multi-user EH-CIoT model in a jamming attack environment. In realistic scenarios, the base station provides wireless communication services to users within its coverage area. Therefore, its service range is the coverage area centered around the base station. Network nodes are usually randomly distributed within the service range of the base station. In addition, according to the 3GPP organizational rules, under the existing 5G background, the coverage radius of macro base stations is over 200 m. Based on the above actual situations, the network size of this paper is set as follows. In the area of 1 km × 1 km, the PBS is located at [500,500]; the ABS is located at [250,250]; the PUs are distributed within a radius of 500 m with the PBS as the center; EH-CIoT nodes and JAN are distributed within a radius of 250 m with the ABS as the center. The users of each node obey the Poisson distribution.

For fair comparison, this paper uniformly considers the channel bandwidth adopted in [27], i.e., 1 MHz. In addition, to verify the effectiveness of the proposed method, this paper considers various types of jamming attacks in existing work [45], including random jamming, scanning jamming, and reactive-scanning jamming. Among them, random jammer randomly chooses a channel to inject jamming signals. The scanning jammer is an improvement based on a random jammer, which can simultaneously randomly jam with multiple channels; thus, it has a greater impact on throughput. Compared to the scanning jammer, the reactive-scanning jammer is more intelligent, so its harm is stronger than the scanning jammer. Considering the energy consumption of the jammer and the effectiveness of the jamming attack, the maximum jamming power of the jammer is usually slightly greater than the transmission power of the EH-CIoT nodes. Therefore, this paper sets the maximum jamming power of the jammer to 0.2W. In addition, this paper compares the proposed LWDDPG resource-allocation algorithm with the Greedy Algorithm, the ACDQN Algorithm in [24], and the DDPG Algorithm in [27].

The simulation uses the Python 3.6 programming language to develop the RL environment, and the results were obtained from the deep learning framework based on TensorFlow [46]. In the simulation, all networks of the LWDDPG-based algorithm have two hidden layers with L1=256 and L2=256 neurons, respectively. To reduce the computational complexity, the activation functions of the hidden layer and the output layer of the critic network and the hidden layer of the participant network are set as the rectified linear units. To limit the range of action, the activation functions of the output layer of the actor network are set to tanh. The optimizer of the critics network and the actors network is Adam [47]. The learning rates of the critic and the actor are set to 0.003. The soft update rate ξ is set to 0.005. The number of maximum episodes is set to 500, and the number of steps per episode is 10∼100. Each episode represents a complete RL process. And a step represents the action performed by the ABS in each episode. The parameters of the neural network are initialized randomly at the beginning of each experiment. At the beginning of each episode, the battery of the EH-CIoT nodes is reset to $E_{max}$. The other simulation parameters are provided in Table 1.

### 8.2. Statistical Results and Analysis

In this subsection, this paper compares the performance of four algorithms, LWDDPG (proposed method), DDPG (method in [27]), ACDQN (method in [24]), and Greedy algorithms under four different environments: no jamming, random jamming, scanning jamming, and reactive-scanning jamming. This paper also briefly analyzes the simulation results.

**Figure 5:**
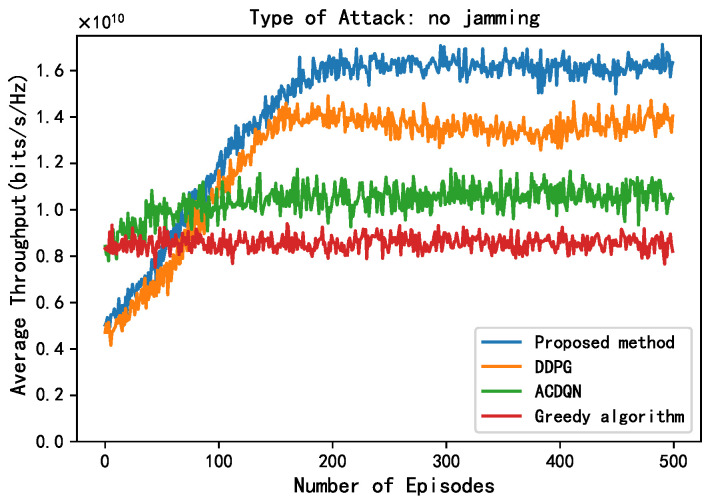
Average throughput vs. episodes under no attack, steps = 100, $B_{max}=1$ J, K=10, N=10, M=3.

First, this paper compares the performance of different methods without jamming. As shown in Figure 5, all the RL algorithms grow with the number of episodes and eventually converge, which proves the convergence of the RL algorithms. The convergence speed of the proposed method is similar to the DDPG algorithm; however, their average throughput after convergence is about 1.62 and 1.4, respectively (for convenience of expression, this paper only uses the mantissa of the scientific notation to express the throughput value of different methods). Obviously, the proposed method is superior to the DDPG algorithm, and the proposed method improves by 15.7% compared to DDPG algorithm. This is because this paper introduces energy rewards to ensure that the ABS always allocates the EH-CIoT nodes with insufficient power to harvest energy on the channels that can obtain more energy, which ensures that the EH-CIoT nodes have sufficient power for transmission in the next timeslot. In addition, the average throughput of the ACDQN algorithm and the Greedy algorithm after convergence is about 1.1 and 0.9, respectively. The proposed method improves by 47.2% and 80% compared to the ACDQN algorithm and the Greedy algorithm. The Greedy algorithm has the worst performance; this is because the decisions of the Greedy algorithm are not based on long-term goals, so it cannot reasonably allocate limited energy to achieve the LTT.

**Figure 6:**
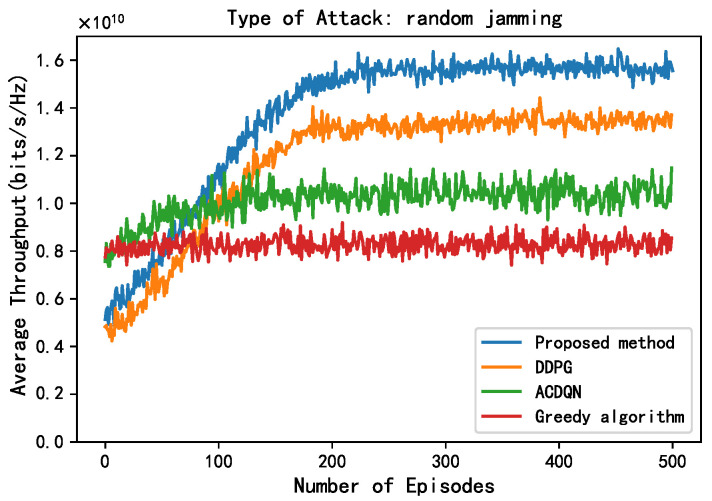
Average throughput vs. episodes under random attack, steps = 100, $B_{max}$ = 1 J, *K* = 10, *N* = 10, *M* = 3.

**Figure 7:**
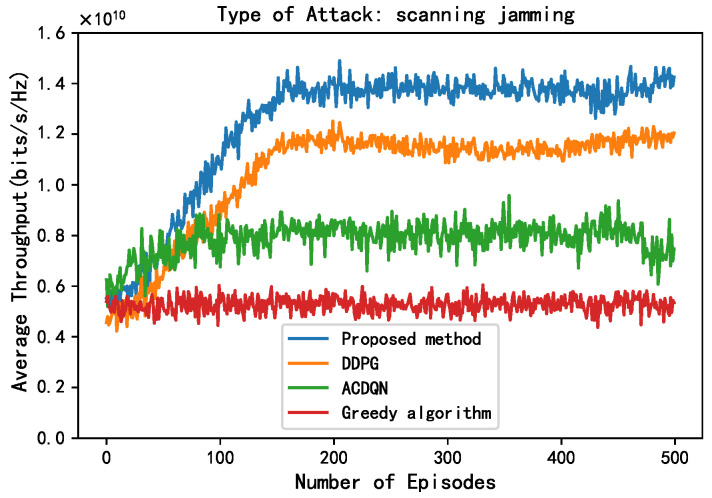
Average throughput vs. episodes under scan attack, steps = 100, $B_{max}$ = 1 J, *K* = 10, *N* = 10, *M* = 3.

As shown in Figure 6 and Figure 7, this paper compares the performance of different methods under random jamming and scanning jamming. Compared to without jamming attack, the convergence throughput of all algorithms decreases under random jamming and scanning jamming attack, and the impact of scanning jamming attacks on throughput is greater. This is because the scanning jamming attack jams multiple channels at once, so it can simultaneously jam the transmission of multiple EH-CIoT nodes, resulting in a greater decrease in throughput of different algorithms. In addition, it can be seen that the convergence throughput of the proposed method is still superior to other algorithms under random jamming and scanning jamming attacks. On the one hand, the proposed method allocates continuous power to the EH-CIoT nodes to achieve more accurate power allocation. And the ABS continuously interacts with the environment to learn, then comprehensively considers the channel jamming, channel gain, and battery power of the EH-CIoT nodes to allocate the EH-CIoT nodes to transmit on the most suitable channels. On the other hand, since the reward function of the proposed method takes energy rewards into account, it can enable the EH-CIoT nodes to have more sufficient power for transmission at each timeslot. Under random jamming attacks, the convergence throughput of the proposed method, the DDPG algorithm, the ACDQN algorithm, and the Greedy algorithm are about 1.59, 1.35, 1, and 0.8, respectively. The proposed method improves by 17.8%, 59%, and 98.8% compared to the DDPG algorithm, the ACDQN algorithm, and the Greedy algorithm. Under scanning jamming attacks, the convergence throughput of the proposed method, the DDPG algorithm, the ACDQN algorithm, and the Greedy algorithm are about 1.4, 1.18, 0.8, and 0.5, respectively. The proposed method improves by 18.6%, 75%, and 180% compared to the DDPG algorithm, the ACDQN algorithm and the Greedy algorithm.

**Figure 8:**
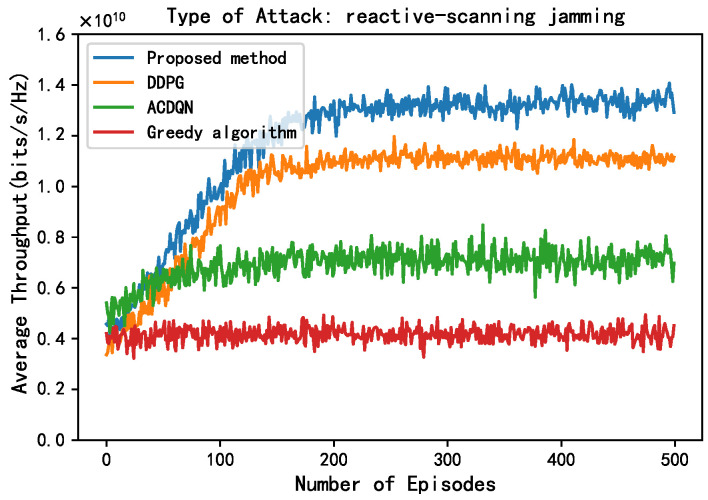
Average throughput vs. episodes under cognitive-scanning attack, steps = 100, $B_{max}$ = 1 J, *K* = 10, *N* = 10, *M* = 3.

In Figure 8, this paper compares the performance of different methods under the reactive-scanning jamming attack. Unlike scanning jamming attacks, in reactive-scanning jamming attacks, the JAN achieves more accurate jamming by sensing ACK/NACK messages in the jammed channel. Therefore, compared to scanning jamming attacks, under reactive-scanning jamming attacks, the convergence throughput of different methods has decreased. Under reactive-scanning jamming attacks, the convergence throughput of the proposed method, the DDPG algorithm, the ACDQN algorithm, and the Greedy algorithm are about 1.38, 1.1, 0.7, and 0.4, respectively. The proposed method improves by 25.4%, 97.1%, and 245% compared to the DDPG algorithm, the ACDQN algorithm, and the Greedy algorithm.

Table 2 shows the percentage decrease in throughput of different methods under different jamming attacks compared to without jamming attacks. The smaller the values corresponding to different methods in Table 2, the better their performance in defending against different jamming attacks. Therefore, from Table 2, it can be intuitively seen that under random jamming attacks, scanning jamming attacks, and reactive-scanning jamming attacks, the throughput decrease percentages of the proposed method are 1.85%, 13.6%, and 14.8%, respectively, which perform the best among all methods. This verifies that the proposed method is more effective than traditional methods in defending against different jamming attacks.

**Figure 9:**
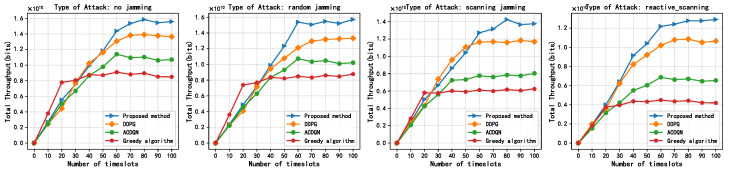
Total throughput vs. steps, episodes = 500, $B_{max}$ = 1 J, *K* = 10, *N* = 10, *M* = 3, γ = 0.9.

Figure 9 shows the relationship between the total throughput of the different methods and the number of timeslots under different jamming situations. As shown in Figure 9, when the battery capacity is fixed at 1 J, the total throughput of all methods gradually increases with timeslots and tends to converge. This is because at the beginning of the timeslots, the ABS knows nothing, or knows inaccurate information about the environment. As timeslots increase, the ABS continuously interacts with the environment to learn and makes great decisions. The difference is that the maximum convergence total throughput of each method is significantly different. Under without jamming attacks, random jamming attacks, scanning jamming attacks, and reactive-scanning jamming attacks, the convergence throughput of the proposed methods are about 1.6, 1.55, 1.4, and 1.3, respectively, and the proposed method is superior to other methods. This is because the proposed method can effectively harvest energy and enable the EH-CIoT nodes to fully utilize limited battery capacity for transmission.

**Figure 10:**
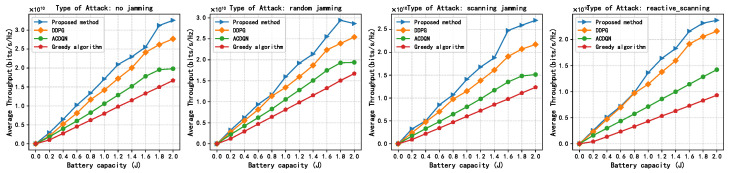
Average throughput vs. max batter capacity, episodes = 500, steps = 100, *K* = 10, *N* = 10, *M* = 3.

Figure 10 shows the relationship between the average throughput of different methods and maximum battery capacity under the different jamming attacks. In Figure 10, the simulation results show that the average throughput of all methods increases with the increase in the maximum battery capacity $B_{\max}$. This is because the larger the battery capacity, the more energy the EH-CIoT nodes can use to transmit, and the EH-CIoT nodes can transmit at a higher power in each time slot. Under reactive-scanning jamming attacks, when the maximum battery capacity is 2 J, the convergence throughput of the proposed method, the DDPG algorithm, the ACDQN algorithm, and the Greedy algorithm are about 2.4, 2.2, 1.4, and 0.93, respectively. The proposed method improves by 9.1%, 71%, and 158% compared to the DDPG algorithm, the ACDQN algorithm, and the Greedy algorithm. When the maximum battery capacity is 1 J, the improvement rates of the proposed method compared to traditional methods are 25.4%, 97.1% and 245%, respectively. Obviously, as the maximum battery capacity increases, the advantages of the proposed method decrease. This indicates that the proposed method is more suitable for the energy-constrained EH-CIoT network.

## 9. Conclusions

This paper proposes an anti-jamming resource-allocation method to maximize the LTT of the EH-CIoT network. First, it models the proposed problem as an MDP without prior knowledge. And the paper proposes a two-dimensional reward vector that includes energy rewards and throughput rewards, so that the ABS can comprehensively consider the energy and throughput of the EH-CIoT nodes, which are closely related to the objective function, and guide the ABS to make optimal decisions. Then, this paper proposes an LWDDPG algorithm to enable the ABS to reasonably allocate transmission channels, continuous power, and work modes to the EH-CIoT nodes through interaction with the environment. Finally, the simulation results demonstrate the validity and superiority of the proposed method, and the proposed method improves by 25.4%, 97.1%, and 245% compared to the DDPG algorithm, the ACDQN algorithm, and the Greedy algorithm.

## Figures and Tables

**Table 1:** Simulation parameters.

Parameters	Value
Number of EH-C nodes *N*	10
Number of primary channels *K*	10
Number of primary users *M*	3
Length of each timeslot *T*	1 s
Control phase duration τ	0.2 s
Path loss exponent α	3.17
Reference distance $d_{0}$	3.3 m
Transmission power $P_{k}^{P}$ of primary user	0.2 W
Maximum transmission power $P_{max}^{C}$ of EH-C nodes	0.1 W
Energy consumed by the handshake $e_{f}$	0.01 J
Noise power *n*	1 × $10^{-10}$ W
Maximum battery capacity $B_{max}$	1 J
Energy-conversion rate η	0.8
Discount rate γ	0.9
SINR threshold $SINR_{threshold}$	5 dB
Capacity *C* of experience buffer *D*	10,000
Minibatch size	256
Antenna length $L_{a}$	0.02 m
Carrier frequency f	900 MHz
Number of channels jammed by scanning attacks KN	2

**Table 2:** Percentage decrease in throughput.

		Method	Proposed Method	DDPG [27]	ACDQN [24]	Greedy
	Percentage					
Jamming Type						
Random Jamming			1.85%	3.57%	9.1%	11.1%
Scanning Jamming			13.6%	15.7%	27.3%	44.5%
Reactive-Scanning			14.8%	21.4%	36.4%	55.6%

## Data Availability

The original contributions presented in the study are included in the article, further inquiries can be directed to the corresponding author.
